# Disinfecting Oven in Mercy Hospital

**Published:** 1876-04

**Authors:** E. Andrews

**Affiliations:** Hospital Surgeon; No. 6, 16th St., Chicago


### DISINFECTING OVEN IN MERCY HOSPITAL.

BY E. ANDREWS, M.D., HOSPITAL SURGEON.

The disinfection of mattresses and pillows in hospitals has been very much neglected. Floors can be scoured, and sheets can be washed, but what can be done with a mattress? Many hospitals have solved the problem by doing nothing. Mattresses which have been occupied by cases of erysipelas, pyæmia, or puerperal fever, were simply laid away to be aired, and then brought into use again, full of deadly fomites for fresh patients—I was about to say victims. By a singular obtuseness, surgeons, who found their wards decimated by pyæmia and erysipelas, forgot their poisoned beds, and gravely asserted that the very stones and bricks of the building were poisoned, and that nothing would do but to erect a new hospital every five years.

Callender, of St. Bartholomew's Hospital, has overthrown this delusion by showing that an old hospital may be managed so as to be safer than any average private house, and that septic influences may be expelled from the building.

But to return to the mattresses. In Mercy Hospital my custom was to place surgical patients on straw mattresses. When a bed was vacated by the recovery or death of the patient, the straw was taken out and burned, and the tick sent to the laundry to be washed. It was then filled with new straw, and laid away ready for use. The pillows were ripped open and the feathers renovated by boiling hot steam, and the pillow ticks washed. By these, and other precautions, disinfection was well accomplished, and pyæmia was almost unknown in the institution. However, the steaming of feathers, the emptying, washing and filling of ticks, and the burning of straw, are troublesome to the attendants, and apt to be neglected, unless constant watchfulness is kept up by the surgeon. To render the disinfection more easy, I have since adopted the plan pursued with so much success in St. Bartholomew's Hospital, London, viz.: the baking of every bed and pillow which has been used by a surgical patient. Dry heat destroys fomites just as effectually as boiling water, and is far more convenient. To accomplish this, the hospital has constructed a simple steam oven on the following plan:

A platform or stratum of parallel steam pipes is made, a little larger than the dimension of a mattress, constituting the foundation. Eighteen inches above this another stratumof pipes is laid, parallel to the first. On the top of the under stratum a floor is laid of galvanized iron, and the whole apparatus enclosed so as to confine the hot air, and convert the space between the strata of steam pipes into a sort of oven. The enclosure has a door the whole length of one side which lets down for the insertion of the articles to be disinfected, and the pipes are connected with the steam boilers which heat the building. When in use, the door is opened, the mattress and pillows are shoved in, the door closed, and the steam, which has a temperature of about 250° Fahrenheit, is let into the pipes. This makes a strong baking heat, and effectually disinfects the beds.

No. 6, 16th St., Chicago.

